# Association Between the CALLY Index and Kidney Stones: Insights From NHANES 2007–2010

**DOI:** 10.1155/mi/9920716

**Published:** 2025-09-30

**Authors:** Liangliang Dai, Chenjie Qiu

**Affiliations:** ^1^Department of Urology, Wujin Hospital Affiliated With Jiangsu University, Changzhou 213004, China; ^2^Department of Urology, The Wujin Clinical College of Xuzhou Medical University, Changzhou 213004, China; ^3^Department of General Surgery, Changzhou Hospital of Traditional Chinese Medicine, Changzhou 213000, China

**Keywords:** CALLY index, immune function, inflammation, kidney stones, NHANES, nutrition

## Abstract

**Background:**

Kidney stones are a prevalent health concern with rising incidence, influenced by factors such as inflammation, nutrition, and immune function. The C-reactive protein (CRP)–albumin–lymphocyte (CALLY) index—a composite measure of CRP, albumin, and lymphocyte count—has demonstrated prognostic value in several cancers. However, its relevance to kidney stone formation remains largely unexplored. This study aimed to examine the association between the CALLY index and kidney stone risk using data from the National Health and Nutrition Examination Survey (NHANES) 2007–2010.

**Methods:**

A total of 10,562 participants were included after applying exclusion criteria. Kidney stone status was determined through self-reported questionnaires, and the CALLY index was calculated accordingly. Associations between the CALLY index and kidney stones were assessed using multivariable logistic regression, restricted cubic spline (RCS) models, and subgroup analyses.

**Results:**

Participants with kidney stones exhibited a lower mean CALLY index. Multivariable analyses revealed a significant inverse association between the CALLY index and kidney stone risk, particularly when the index was modeled categorically. Individuals in the highest quartile of the CALLY index had a 27.3% lower risk of kidney stones compared with those in the lowest quartile. RCS analysis further confirmed a nonlinear relationship.

**Conclusion:**

The findings suggest that the CALLY index is negatively associated with kidney stone risk.

## 1. Introduction

Kidney stone formation arises from the accumulation of abiotic substances, such as salt crystals, together with biomolecules, including urinary macromolecules, within renal tissue or the urinary tract [1]. In the United States, the prevalence of kidney stones has increased markedly, from 3.2% during 1976–1980 to 5.2% in 1988–1994 and further to 8.8% between 2007 and 2010 [2, 3]. A similar upward trend has been documented worldwide [1, 4–7]. Kidney stones are also characterized by a high recurrence rate: approximately 35%–50% of patients experience recurrence within 5 years of the initial episode, and nearly half develop a second episode within 10 years, often accompanied by progressive renal function decline [8]. This condition places a substantial burden on public healthcare systems. Around the year 2000, annual healthcare expenditures for kidney stones in the U.S. exceeded 2 billion dollars, with projections suggesting these costs may double by 2030 [9, 10]. Moreover, patients with symptomatic kidney stones face an increased risk of chronic kidney disease (CKD) and progression to end-stage renal disease [11]. Stone formation is influenced by a range of factors, including dietary patterns, genetic susceptibility, systemic conditions (e.g., metabolic syndrome, coronary artery disease [CAD], and CKD), medication use, and physical inactivity [12–16]. Early recognition and management of these risk factors are critical to alleviating the healthcare burden and improving long-term patient outcomes.

Kidney stone formation is a multifactorial process influenced by inflammation, nutritional status, and immune function. Inflammation is a key driver of stone pathogenesis. *C*-reactive protein (CRP), a widely used clinical marker of systemic inflammation, has been consistently linked to an elevated risk of kidney stones [17]. Higher serum CRP levels are positively associated with self-reported kidney stone incidence, particularly among younger individuals, suggesting that systemic inflammation contributes to stone development [18, 19]. Nutritional status also plays an essential role. While adequate fluid intake generally confers protection, the effects of specific beverages remain under investigation. Dietary factors—including protein, carbohydrates, oxalates, calcium, and sodium chloride—exert differential influences on stone risk [20]. Notably, animal protein consumption increases the likelihood of stone formation, whereas plant protein appears protective [21, 22]. Albumin, a biomarker of nutritional status, has likewise been shown to exert a preventive effect [23]. Immune function further shapes susceptibility to stone disease. Experimental studies have demonstrated upregulation of inflammatory, immune, and complement activation pathways in both animal models and renal tissue from patients with kidney stones [24]. Mendelian randomization analyses have clarified causal roles of immune factors, identifying both protective and deleterious effects of specific immune cell populations [25]. Lymphocytes, in particular, play a central role, with composite indices such as the neutrophil-to-lymphocyte ratio (NLR) and the systemic immune-inflammation index (SII) increasingly applied as indicators in kidney stone research [26, 27].

The CRP–albumin–lymphocyte (CALLY) index is an emerging biomarker with demonstrated prognostic value across multiple malignancies [28]. It combines three key components: inflammatory status (CRP), nutritional status (albumin), and immune function (lymphocytes). While inflammation is an essential defense mechanism, persistent low-grade inflammation increases susceptibility to chronic diseases such as cancer and cardiovascular disorders. The CALLY index has shown particular utility in colorectal cancer, where it outperforms conventional prognostic factors [29–31]. It has also been validated as a noninvasive predictor of posthepatectomy outcomes in hepatocellular carcinoma [28, 32] and as a reliable indicator of overall survival in patients with non-small cell lung cancer and esophageal cancer, thereby guiding clinical decision-making [33–36]. Beyond oncology, the index has demonstrated relevance in cardiovascular diseases, including myocardial infarction and CAD [37–39]. Despite its broad applications, evidence on the role of the CALLY index in kidney stone disease remains scarce [40]. To address this gap, the present study utilizes data from the National Health and Nutrition Examination Survey (NHANES), a large-scale public health database, to explore the association between the CALLY index and kidney stone risk.

## 2. Materials and Methods

### 2.1. Study Population

The NHANES is a large-scale survey program conducted by the National Center for Health Statistics (NCHS) under the Centers for Disease Control and Prevention (CDC) in the United States. It consists of two major components: (1) structured interviews that collect demographic, socioeconomic, dietary, and health-related information and (2) standardized physical examinations performed by trained medical personnel, including clinical assessments, physiological measurements, and laboratory tests. Data collected span demographic, dietary, examination, laboratory, and questionnaire domains. NHANES findings are widely used to monitor disease prevalence, identify risk factors, and evaluate the relationships among nutrition, health promotion, and disease prevention. Written informed consent is obtained from all participants prior to interviews and examinations. The survey protocol is approved by the NCHS Institutional Review Board; therefore, no additional ethical approval was required for the present study.

**Figure 1:**
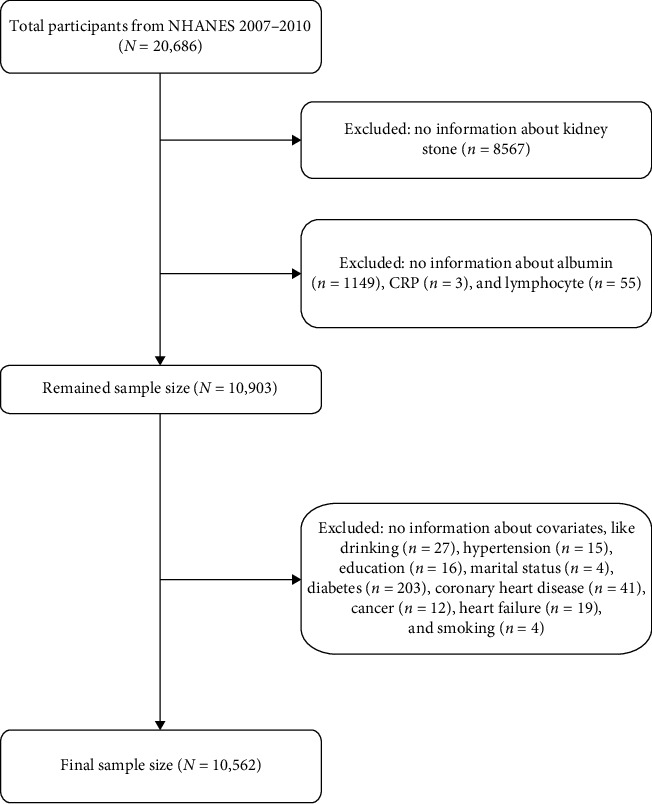
Flow chart of the participants selection. NHANES, National Health and Nutrition Examination Survey.

For this analysis, we used data from two NHANES cycles (2007–2010), comprising 20,686 participants. Exclusion criteria were (1) missing kidney stone outcome data; (2) missing data on CALLY index components (albumin, CRP, and lymphocytes); and (3) incomplete covariate information, including alcohol consumption, hypertension, education level, marital status, diabetes, coronary heart disease, heart failure, cancer, and smoking status. After applying these criteria, 10,562 participants were included in the final analysis (Figure 1). To address missing covariate data, we used the “mice” package to perform multiple imputation.

### 2.2. Data Collection and Definition

In this study, the CALLY index was used as the primary exposure variable to evaluate participants' inflammation, nutritional status, and immune function. The index was calculated using the following formula, $\text{CALLY index}=\frac{\text{Albumin }\times \text{ Lymphocyte}}{\left(\text{CRP }\times \;10\right)}$. Kidney stone status was determined through a standardized questionnaire that asked participants whether they had ever been diagnosed with kidney stones. A “yes” response indicated a history of kidney stones, whereas a “no” response indicated no history. The validity of self-reported kidney stone status has been confirmed in prior studies [41].

Potential confounders associated with both the CALLY index and kidney stone risk were identified using NHANES data. Demographic factors are as follows: sex (male and female), age (≤60 years and > 60 years), race (Mexican American, other Hispanic, non-Hispanic White, non-Hispanic Black, and other), education (below high school, high school, and above high school), marital status (living with partner and living alone), income-to-poverty ratio (PIR :   ≤ 1.3, 1.3 − 3.5,  and > 3.5), and body mass index (BMI)(BMI :   ≤ 18.5 [emaciation],  18.5 − 25 [normal],  25 − 30 [overweight],  and > 30 [obesity]). Medical history is as follows: self-reported hypertension, diabetes, coronary heart disease, heart failure, and cancer. Lifestyle factors: Smoking status was categorized as never, former, or current smoker. Participants who reported smoking fewer than 100 cigarettes in their lifetime were classified as never smokers. Those who reported smoking ≥ 100 cigarettes were further categorized: individuals who currently smoked “every day” or “some days” were considered current smokers, while those who reported not smoking at all were defined as former smokers. Alcohol consumption was divided into three groups: nondrinkers (fewer than 12 drinks per year), mild drinkers (≤1 drink/day for women or ≤2 drinks/day for men), and severe drinkers (exceeding these thresholds within the past year). To improve analytical accuracy, several laboratory indicators were also included: urinary creatinine, blood urea nitrogen, blood calcium, serum creatinine, serum phosphorus, uric acid, white blood cell count, monocyte count, neutrophil count, and platelet count.

### 2.3. Statistical Analysis

In this study, baseline characteristic and correlation analyses were performed using NHANES data, with sample weights applied to account for the survey's stratified, multistage sampling design. Baseline characteristics were stratified by kidney stone status. Continuous variables were presented as weighted means ± standard deviations (SDs) and categorical variables as frequencies with weighted percentages. Group comparisons were conducted using weighted t-tests for continuous variables and chi-squared tests for categorical variables.

The association between the CALLY index and kidney stones was evaluated using weighted multivariable logistic regression. The CALLY index was examined both as a continuous variable and as quartiles. Three regression models were constructed: (1) Crude model, with no covariate adjustment; (2) Model 1, adjusted for sex, age, race, and education; and (3) Model 2, further adjusted for marital status, PIR, hypertension, diabetes, coronary heart disease, heart failure, cancer, smoking status, alcohol consumption, and BMI. Restricted cubic spline (RCS) analysis was applied to assess potential nonlinear relationships. Subgroup analysis is mainly based on 14 clinical indicators, including demographics, comorbidities, and lifestyle, all of which can be obtained from the NHANES database. The interaction we conducted was evaluated by adding a product term (i.e., CALLY index multiplied by stratified variables) to the regression model and testing its *p*-value. At the same time, multiple comparisons were added in the interaction testing using the FDR method.

All statistical analyses and visualizations were performed using R software. A two-sided *p*-value < 0.05 was considered statistically significant.

## 3. Results

### 3.1. Participant Characteristics

This cross-sectional study included 10,562 participants. Baseline characteristics, stratified by kidney stone history, are presented in Table 1. The weighted mean age of the study population was 46.74 years (SD = 16.62), and 48.2% (*n* = 5135) were male. The overall weighted mean CALLY index was 11.97 (SD = 21.31). Participants with a history of kidney stones had a lower mean CALLY index (9.12, SD = 14.23) than those without kidney stones (12.25, SD = 21.86). In total, 957 individuals (8.9% of the population) reported a history of kidney stones. Compared with participants without stones, those with kidney stones were more likely to be male, older, non-Hispanic White, and living with a partner. They also exhibited higher prevalence of hypertension, diabetes, heart failure, coronary heart disease, and cancer. With respect to lifestyle factors, former smokers were more common among individuals with kidney stones, whereas severe drinkers were less prevalent. Participants with kidney stones also had higher BMI values, with a larger proportion categorized as overweight or obese. Laboratory findings further distinguished the two groups. Individuals with kidney stones had elevated levels of urinary creatinine, blood urea nitrogen, serum creatinine, uric acid, white blood cells, monocytes, and neutrophils, while showing lower levels of albumin and phosphorus.

### 3.2. Association of CALLY Index and Kidney Stones

**Figure 2:**
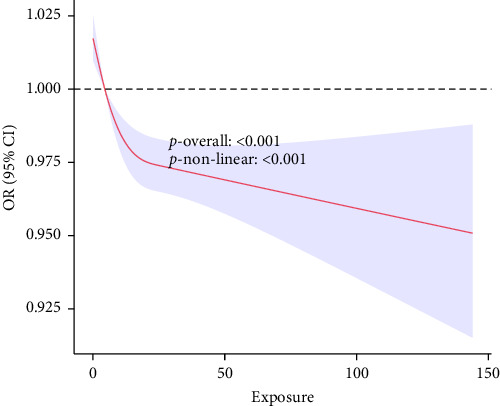
The exposure-response association of the CALLY index with the prevalence of kidney stones by restricted cubic spline (RCS). The red and purple lines represent the estimated values and corresponding 95% confidence interval, respectively. The adjustment factors included age (≤ 60 and > 60), gender (male and female), race (Mexican American, non-Hispanic Black, non-Hispanic White, other Hispanic, other race), education (below high school, high school, and above high school), marital status (living alone and with partner), PIR (≤ 1.3, 1.3–3.5, >3.5), BMI (emaciation, normal, overweight, and obesity), hypertension (no and yes), diabetes (no and yes), heart failure (no and yes), coronary heart disease (no and yes), cancer (no and yes), drinking (never, mild, and severe), and smoking (never, former, and now).

Multivariable logistic regression analysis demonstrated a significant inverse association between the CALLY index and kidney stone risk (Table 2). When treated as a continuous variable, the CALLY index showed a trend toward reduced kidney stone risk in Model 2 (OR=0.995 and *p*=0.086), though this did not reach statistical significance. However, when analyzed categorically, participants in the highest CALLY quartile had a 27.3% significantly lower risk of kidney stones (OR = 0.727, 95% CI: 0.582–0.907, *p*=0.030), indicating a dose-dependent protective effect. RCS analysis further confirmed a nonlinear association between the CALLY index and kidney stone risk (Figure 2, nonlinear *p*  < 0.001). These findings suggest that higher CALLY index levels may be protective against kidney stone formation.

### 3.3. Subgroup Analysis

**Figure 3:**
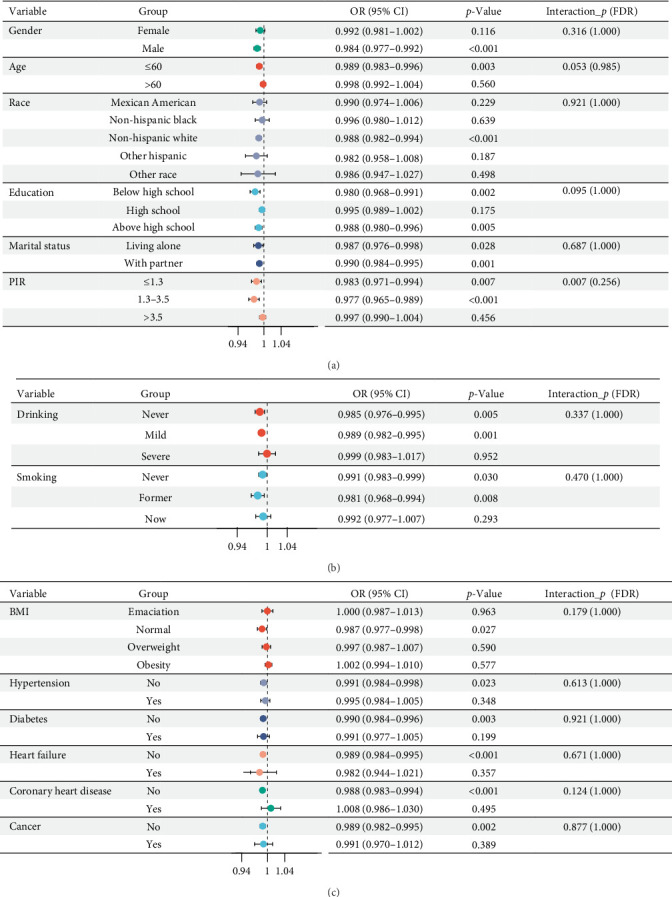
Subgroup analysis for the association of the CALLY index with kidney stones. All covariates (as in Model 2) were adjusted except the stratification variable itself. (A) Demographics. (B) Lifestyle. (C) Comorbidities.

This study further explored the relationship between the CALLY index and kidney stones through subgroup analysis. The subgroup analysis results indicate that the protective effect of CALLY index on the risk of kidney stones is particularly significant in the following groups: males, age ≤ 60 years, non-Hispanic whites, below high school or above high school, PIR ≤ 3.5, without hypertension, diabetes, heart failure, coronary heart disease, or cancer, never smokers and former smokers, nondrinkers and mild drinkers, and normal BMI participants. Meanwhile, no significant interactions were observed across all subgroups. The impact of CALLY index on the risk of kidney stones is not affected by these clinical indicators (Figure 3, FDR > 0.05). It is worth noting that the unadjusted interaction *p*-value of PIR is significant, but it is no longer significant after FDR correction, indicating that economic level may regulate the strength of protective effects, but more evidence is still needed to support it.

## 4. Discussion

This study utilized the NHANES database to perform a cross-sectional analysis investigating the association between the CALLY index and kidney stone occurrence. Our results demonstrated a significant inverse relationship, with individuals reporting kidney stones exhibiting lower CALLY index values. Multivariable logistic regression indicated that, when treated as a continuous variable, higher CALLY index levels were associated with a reduced risk of kidney stones; however, this association was no longer statistically significant in Model 2, which adjusted for all covariates. When analyzed categorically, participants in Quartiles 3 and 4 consistently showed a significantly lower risk of kidney stones compared with Quartile 1 across all models, with a clear downward trend. RCS analysis confirmed a nonlinear inverse association between the CALLY index and kidney stone occurrence. Subgroup analyses further demonstrated that this negative correlation was generally consistent across stratified groups.

Current research on the CALLY index has primarily focused on oncological conditions, including tumor incidence, prognosis, recurrence, and complication prediction [31, 42–44]. However, its potential applicability in nononcological diseases remains underexplored. To our knowledge, this study is the first to investigate the relevance of the CALLY index in the context of kidney stones. Although this analysis is retrospective and cross-sectional, it provides important clinical insights for future research on the role of the CALLY index in nononcological conditions. For example, Xu et al. [45] reported a significant inverse association between the CALLY index and the risk of cardiorenal syndrome, indicating that lower CALLY index values were linked to higher susceptibility and demonstrating a clear trend. Similarly, Ji et al. [37] found that higher CALLY index levels reduced the risk of both short- and long-term major adverse cardiac events (MACEs) in patients with ST-segment elevation myocardial infarction (STEMI) and lowered the likelihood of severe CAD in this population. Furthermore, other studies have shown that decreased CALLY index values are associated with poorer prognostic outcomes in patients with CAD following percutaneous coronary intervention (PCI) or among elderly individuals, including increased all-cause and cardiac mortality, as well as higher rates of major adverse cardiovascular and cerebrovascular events [38, 39].

Inflammation is a key contributor to kidney stone formation, with multiple inflammatory processes implicated in pathogenesis, including the production of reactive oxygen species, activation of inflammasomes, and upregulation of molecules involved in inflammatory cascades [24]. Macrophages play a central role: proinflammatory M1 macrophage activation is associated with stone formation, whereas anti-inflammatory M2 macrophages appear to inhibit this process. Notably, genes related to M2 macrophages have been linked to suppression of stone formation [46, 47]. CRP is a well-established inflammatory biomarker and a crucial regulator of host defense against infections, tissue injury, and autoimmune diseases, with levels rising sharply at sites of inflammation [48]. Mao et al. [26] demonstrated that a high NLR is associated with an increased prevalence of kidney stones and a higher number of stone passages, indicating that elevated neutrophils and reduced lymphocytes correlate with greater stone risk. This aligns with our findings, which identify higher lymphocyte counts as protective against stone formation. Furthermore, individuals with uric acid–rich stones and elevated NLR levels are at increased risk of developing CKD [49]. Dietary protein intake exhibits complex associations with kidney stone risk. Nondairy animal protein has been linked to a modestly increased risk in men but not in women, whereas dairy protein appears protective in young women [21]. However, the overall impact of protein on stone formation remains inconclusive. For example, Reddy et al. [50] reported that low-carbohydrate, high-protein diets (>2 g/kg/day) increased urinary calcium excretion, whereas Tracy et al. [51] found no significant differences in urinary calcium across various protein sources, including fish, beef, and chicken. Albumin, one of the most abundant urinary proteins, acts as a potent nucleator of calcium oxalate crystals in vitro. Its nucleating effect primarily promotes the formation of calcium oxalate dihydrate crystals, whereas monohydrate crystals—the core component of most calcium oxalate stones—form in its absence. Both crystal forms are present in healthy individuals and stone formers, but the monohydrate form is exclusively observed in stone formers. These findings suggest that serum albumin may stabilize urinary crystallization by favoring dihydrate formation, potentially offering a protective effect against stone formation [52]. The CALLY index synergistically reflects inflammation (CRP), nutrition (albumin), and immunity (lymphocytes)—all implicated in stone pathogenesis. Elevated CRP promotes oxidative stress and inflammasome activation, fostering crystal aggregation [24, 46]. Low albumin reduces inhibitory proteins like Tamm–Horsfall glycoprotein, weakening defense against crystallization [23, 52]. Lymphopenia reflects impaired immune surveillance, permitting inflammatory cascades that aid stone growth [26]. Thus, the CALLY index may capture a triad of dysregulations that collectively increase stone susceptibility.

This study is the first to examine the association between the CALLY index and kidney stones using the NHANES database, highlighting its potential utility in evaluating nonneoplastic diseases. Nevertheless, several limitations should be acknowledged. First, the retrospective design of this analysis introduces inherent biases, and the completeness and accuracy of kidney stone data collected by NHANES are constrained, as they rely entirely on self-reported questionnaires. Self-reporting of kidney stones may underestimate the prevalence of kidney stones, especially ignoring asymptomatic cases, which may affect the results of this study. Although the accuracy of self-reported kidney stones in NHANES is still unclear, several previous studies have validated its reliability in other populations [53–55]. Second, although multiple covariates were included, the range of factors associated with kidney stone risk is not exhaustive, potentially influencing the results of the multivariable logistic regression. Finally, while our findings demonstrate an inverse correlation between the CALLY index and kidney stone occurrence, causality cannot be inferred. Therefore, these observations require validation through large-scale prospective studies.

## 5. Conclusion

Using NHANES data, this study demonstrated an inverse association between the CALLY index and kidney stone incidence. Due to its clinical accessibility, the CALLY index may represent a valuable tool for early identification of individuals at risk for kidney stones, thereby potentially reducing the associated healthcare burden.

## Figures and Tables

**Table 1:** Weighted characteristics of the study population based on kidney stones.

Variable	Overall (10,562)	No (9605, 91.1%)	Yes (957, 8.9%)	*p*-Value
Gender (%)	—	—	—	<0.001
Female	5427 (51.8)	5036 (53.0)	391 (39.9)	—
Male	5135 (48.2)	4569 (47.0)	566 (60.1)	—
Age (years, continuous)	46.74 (16.62)	46.09 (16.57)	53.33 (15.69)	<0.001
Age (categories)	—	—	—	<0.001
≤60 years	7242 (77.9)	6726 (79.2)	516 (65.5)	—
>60 years	3320 (22.1)	2879 (20.8)	441 (34.5)	—
Race (%)	—	—	—	<0.001
Mexican American	1894 (8.5)	1767 (8.8)	127 (5.0)	—
Non-Hispanic Black	1911 (10.5)	1808 (11.0)	103 (5.2)	—
Non-Hispanic White	5121 (69.6)	4529 (68.5)	592 (81.6)	—
Other Hispanic	1130 (5.0)	1023 (5.0)	107 (4.7)	—
Other Race	506 (6.4)	478 (6.7)	28 (3.6)	—
Education (%)	—	—	—	0.204
Below high school	3083 (19.1)	2798 (19.1)	285 (19.2)	—
High school	2508 (23.9)	2273 (23.7)	235 (26.1)	—
Above high school	4971 (57.0)	4534 (57.2)	437 (54.7)	—
Marital status (%)	—	—	—	0.008
Living alone	4174 (35.5)	3831 (36.0)	343 (30.3)	—
With partner	6388 (64.5)	5774 (64.0)	614 (69.7)	—
PIR (continuous)	3.00 (1.65)	3.00 (1.65)	2.99 (1.59)	0.95
PIR (categories)	—	—	—	0.04
≤1.3	3459 (21.4)	3167 (21.7)	292 (18.5)	—
>3.5	3098 (42.9)	2814 (43.0)	284 (41.1)	—
1.3–3.5	4005 (35.7)	3624 (35.2)	381 (40.4)	—
Urine creatinine, µmol/L	10,602.81 (6806.23)	10,555.27 (6855.33)	11,087.92 (6265.96)	0.025
Drinking (%)	—	—	—	0.002
Never	2968 (23.9)	2679 (23.6)	289 (26.6)	—
Mild	6593 (65.8)	5988 (65.7)	605 (67.1)	—
Severe	1001 (10.3)	938 (10.7)	63 (6.4)	—
Smoking (%)	—	—	—	0.002
Never	5647 (54.5)	5191 (54.9)	456 (49.7)	—
Former	2584 (24.0)	2270 (23.4)	314 (30.2)	—
Now	2331 (21.5)	2144 (21.7)	187 (20.0)	—
Blood albumin, g/dL	4.27 (0.34)	4.27 (0.34)	4.23 (0.33)	0.002
Blood urea nitrogen, mmol/L	4.67 (1.91)	4.62 (1.88)	5.19 (2.19)	<0.001
Blood calcium, mmol/L	2.36 (0.09)	2.36 (0.09)	2.36 (0.09)	0.669
Blood creatinine, umol/L	77.62 (29.14)	77.09 (27.13)	83.10 (44.41)	<0.001
Blood phosphorus, mmol/L	1.22 (0.18)	1.22 (0.18)	1.19 (0.18)	<0.001
Blood uric acid, umol/L	324.63 (84.80)	322.75 (84.15)	343.74 (89.02)	<0.001
BMI (kg/m^2^, continuous)	28.60 (6.69)	28.46 (6.67)	30.02 (6.78)	<0.001
BMI (categories)	—	—	—	<0.001
Emaciation	165 (1.7)	158 (1.8)	7 (0.7)	—
Normal	2887 (30.1)	2694 (31.0)	193 (20.9)	—
Overweight	3633 (34.1)	3296 (33.9)	337 (35.8)	—
Obesity	3877 (34.1)	3457 (33.3)	420 (42.5)	—
Hypertension (%)	—	—	—	<0.001
No	6940 (70.7)	6459 (72.2)	481 (55.2)	—
Yes	3622 (29.3)	3146 (27.8)	476 (44.8)	—
Diabetes (%)	—	—	—	<0.001
No	9295 (91.6)	8538 (92.4)	757 (83.8)	—
Yes	1267 (8.4)	1067 (7.6)	200 (16.2)	—
Heart failure (%)	—	—	—	<0.001
No	10,253 (97.9)	9353 (98.2)	900 (95.2)	—
Yes	309 (2.1)	252 (1.8)	57 (4.8)	—
Coronary heart disease (%)	—	—	—	<0.001
No	10,140 (96.8)	9265 (97.3)	875 (92.6)	—
Yes	422 (3.2)	340 (2.7)	82 (7.4)	—
Cancer (%)	—	—	—	0.001
No	9551 (90.7)	8731 (91.1)	820 (87.1)	—
Yes	1011 (9.3)	874 (8.9)	137 (12.9)	—
WBC, 1000 cells/µL	7.22 (2.26)	7.20 (2.22)	7.41 (2.60)	0.037
Lymphocyte, 1000 cells/µL	2.13 (0.92)	2.14 (0.93)	2.09 (0.79)	0.203
Monocyte, 1000 cells/µL	0.55 (0.19)	0.55 (0.19)	0.57 (0.18)	0.020
Neutrophil, 1000 cells/µL	4.29 (1.73)	4.27 (1.69)	4.49 (2.11)	0.006
Platelet, 1000 cells/µL	253.94 (66.28)	254.31 (65.70)	250.22 (71.89)	0.154
CRP, mg/dL	0.38 (0.73)	0.38 (0.73)	0.43 (0.75)	0.112
CALLY index	11.97 (21.31)	12.25 (21.86)	9.12 (14.23)	<0.001

Abbreviations: BMI, body mass index; CALLY, C-reactive protein-albumin-lymphocyte; CRP, C-reactive protein; PIR, income-to-poverty ratio; WBC, white blood cell.

**Table 2:** The association of the CALLY index with kidney stones.

Exposure	Crude model^a^		Model 1^b^		Model 2^c^	
	OR (95% CI)	*p*-Value	OR (95% CI)	*p*-Value	OR (95% CI)	*p*-Value
CALLY index (continuous)	0.989 (0.983–0.994)	<0.001	0.990 (0.984–0.996)	0.002	0.995 (0.990–1)	0.086
CALLY index (categories)						
Q1	Reference	—	Reference	—	Reference	—
Q2	0.917 (0.738–1.140)	0.441	0.878 (0.700–1.101)	0.271	0.963 (0.773–1.199)	0.747
Q3	0.705 (0.572–0.870)	0.003	0.658 (0.526–0.824)	0.002	0.754 (0.604–0.940)	0.046
Q4	0.587 (0.486–0.708)	<0.001	0.576 (0.460–0.723)	<0.001	0.727 (0.582–0.907)	0.030
*p* for trend	<0.001	—	<0.001	—	<0.001	—

^a^Crude model was unadjusted for any covariates.

^b^Model 1 was adjusted for age (≤ 60 and >60), gender (male and female), race (Mexican American, non-Hispanic Black, non-Hispanic White, other Hispanic, and other race), and education (below high school, high school, and above high school).

^c^Model 2 was adjusted for age (≤ 60 and >60), gender (male and female), race (Mexican American, non-Hispanic Black, non-Hispanic White, other Hispanic, and other race), education (below high school, high school, and above high school), marital status (living alone and with partner), PIR (≤ 1.3, 1.3–3.5, >3.5), BMI (emaciation, normal, overweight, and obesity), hypertension (no and yes), diabetes (no and yes), heart failure (no and yes), coronary heart disease (no and yes), cancer (no and yes), drinking (never, mild, and severe), and smoking (never, former, and now).

## Data Availability

All original contributions presented in this study are contained within the article. Further information or clarifications can be obtained from the corresponding author upon request.
